# Enhanced Thermal Conductivity and Dielectric Properties of Epoxy Composites with Fluorinated Graphene Nanofillers

**DOI:** 10.3390/nano13162322

**Published:** 2023-08-12

**Authors:** Jiacheng Zhang, Zi Wang, Guoqing Jiang, Huachao Wei, Zongxi Zhang, Junwen Ren

**Affiliations:** 1 College of Electrical Engineering, Sichuan University, Chengdu 610065, China; zhangjiacheng1@stu.scu.edu.cn (J.Z.); zwang0705@stu.scu.edu.cn (Z.W.); jiangguoqing@stu.scu.edu.cn (G.J.); weihuachao@stu.scu.edu.cn (H.W.); 2 Electric Power Research Institute, State Grid Corporation of Sichuan Province, Chengdu 610072, China; bianchao@stu.scu.edu.cn

**Keywords:** epoxy, fluorinated graphene, composites, thermal conductivity, dielectric properties

## Abstract

The demand for high-performance dielectrics has increased due to the rapid development of modern electric power and electronic technology. Composite dielectrics, which can overcome the limitations of traditional single polymers in thermal conductivity, dielectric properties and mechanical performance, have received considerable attention. In this study, we report a multifunctional nanocomposite material fabricated by blending fluorinated graphene (F-graphene) with epoxy resin. The F-graphene/epoxy composite exhibited a high thermal conductivity of 0.3304 W·m^−1^·K^−1^ at a low filler loading of 1.0 wt.%, which was 67.63% higher than that of pure epoxy. The composite dielectric also showed high breakdown strength (78.60 kV/mm), high dielectric constant (8.23), low dielectric loss (<0.015) and low AC conductivity (<10^−11^ S·m^−1^). Moreover, the composite demonstrated high thermal stability and strong mechanical strength. It is believed that the F-graphene/epoxy composite has outstanding performance in various aspects and can enable the development and manufacturing of advanced electric power and electronic equipment devices.

## 1. Introduction

Polymer dielectrics have extensive applications in advanced electronic devices and power systems because of their characteristics of electrical insulation, easy processing and low density [1,2,3,4,5]. However, with the development of modern electronic devices towards miniaturization, high speed, and high frequency, and the increase in voltage level and deterioration of power quality in power systems, the low thermal conductivity of polymer dielectrics can no longer satisfy the growing need for efficient heat dissipation [6,7,8,9,10,11]. A promising way to enhance the thermal conductivity of materials is to form composites by incorporating high thermal conductivity fillers into the polymer matrix [8,10,12,13]. Various fillers such as silver, Al_2_O_3_, boron nitride and carbon nanotubes have been applied to improve the thermal conductivity of polymer dielectrics, and satisfactory results have been obtained [14,15,16,17,18]. Among the currently known thermal conductive fillers, graphene (GR) and its derivatives have a thermal conductivity higher than any other one (over 5000 W·m^−1^·K^−1^), and thus they are considered to be the most promising filler for enhancing the thermal performance of polymer dielectrics [6,19]. Zhang et al.’s finding indicates that adding 0.1 wt.% of graphene to the composite can increase its thermal conductivity to 1.6 times that of pure epoxy resin and simultaneously enhance the antistatic performance [20]. Furthermore, it is of interest to note that it has been proven that the effect of carbon nanofillers on the thermal conductivity, physicochemical and mechanical properties of epoxy polymer composites can be determined by many factors, such as filler compatibility, dispersion orientation of the filler, structure of the filler, etc. For instance, Wang et al. fabricated a thermally conductive epoxy composite by incorporating three-dimensional (3D) polydopamine-graphene foam (PGF) with good interfacial bonding into epoxy resin. The resulting composite exhibited a thermal conductivity of 1.05 W·m^−1^·K^−1^ at 3.02 vol% filler loading, which was 4.3 times higher than that of neat epoxy resin [21]. Lin et al. grafted amino groups on graphene oxide (GO) to improve its interfacial compatibility with epoxy and reduced the covalently modified GO to repair the defects [22]. The epoxy composites produced with improved interfacial compatibility had improved thermal stability, significantly enhanced thermal conductivity (by 650%) and remained well insulated. To realize the oriented dispersion of carbon nanofillers, some scholars have also applied graphene in conjunction with other magnetic fillers, which can further enhance the thermal conductivity of the composite [4]. For instance, Wu et al. combined magnetic Fe_3_O_4_ with graphene and realized the orientation alignment of GR-Fe_3_O_4_ in an epoxy matrix using a magnetic field. When the GR content was 0.134 wt.%, the thermal conductivity of the composite was 54.8% higher than that of pure epoxy. Meanwhile, the product’s thermal stability and dynamic thermomechanical properties were enhanced [23]. Hu et al. used nickel nanoparticles to modify graphene oxide carbon nanotubes so that they can be vertically oriented under the effect of an applied weak magnetic field when doped into an epoxy matrix [24]. The thermal conductivity of the composite is increased to 2.67 times that of the pure epoxy while a significant limiting effect on the expansion of the material in the vertical direction is observed. In improving the structure of carbon nanofillers, Wang et al. prepared long-range ordered carbon/graphene/MgO ternary foam (CGMF) and observed an excellent in-plane thermal conductivity of 4.87 W·m^−1^·K^−1^ at 12.96 vol% filler doping after compositing with an epoxy matrix, while the composite maintained strong electrical insulation performance [25]. Mostovoy et al. used electrochemical oxidized graphite powder to obtain thermally expanded graphene by thermal exfoliation, and the worm-like structure of thermally expanded graphene has a large interlayer distance and a highly activated and branched surface [26]. The prepared epoxy polymer composites exhibit high thermal stability, strong crack resistance and flame retardancy, and the thermal conductivity is improved up to 4.3 times. Yet, these strategies of modification or orientation arrangement of fillers, while realizing the enhancement of thermal conductivity, physicochemical and mechanical properties, use nitric acid, amine and other substances in the process, and the complex preparation process also brings about environmental concerns and production difficulties.

In addition to high thermal conductivity as well as good physicochemical and mechanical properties, polymer dielectrics also require strong insulation and outstanding dielectric performance, especially for applications such as power electronic device packaging, insulation, support, etc. However, graphene as a carbon material has high electrical conductivity, which hinders its application in scenarios that demand high insulation and low dielectric loss [27]. In this context, fluorinated graphene (F-graphene), a graphene derivative, has been given great expectations for its excellent properties such as high thermal conductivity, wide band gap (3.0–4.2 eV), low electrical conductivity (10^−11^ S/cm) and high stability, which is mainly due to the invasion of fluorine that transforms the structure of C-C bonds from sp^2^ to sp^3^ [28,29,30,31,32,33]. For example, Yang et al. fabricated polymer nanodielectrics employing F-graphene and polyvinylidene fluoride (PVDF) and found that adding 0.2 wt.% of F-graphene could enhance the breakdown strength (*E_b_*) by 39.4% and simultaneously reduce the dielectric loss [34]. Zhang et al. developed a fluorinated graphene aerogel (FGA), which was combined with epoxy resin to observe a low electrical conductivity of 10^−9^ S/m and a high thermal conductivity of 3.57 W·m^−1^·K^−1^ [27]. Mani et al. impregnated polyurethane foam coated with F-graphene with epoxy resin and obtained a composite with a thermal conductivity improvement of 51 times compared to neat epoxy and electrical insulation (10^9^ Ω/m) [35]. In addition, the related literature also reported the enhancement of mechanical properties such as the stress–strain of the composites after the incorporation of F-graphene [27,35,36].

Here, we report a method of preparing F-graphene/epoxy composites with high thermal conductivity and outstanding dielectric properties by blending fluorinated graphene as a nanofiller with epoxy resin. Systematic studies were carried out on the thermal conductivity, dielectric and mechanical properties of the obtained F-graphene/epoxy composites. The results show that the introduction of F-graphene enhances the thermal conductivity of the epoxy resin matrix while exhibiting synergistic improvement in dielectric properties, insulation properties, mechanical strength and other aspects. The preparation method of blending F-graphene nanofillers with an epoxy matrix also introduces the practical advantages of a simple and non-polluting process. We believe that the composite developed in this work can provide new options for future power electronic device insulation, packaging and other needs.

## 2. Experimental Section

### 2.1. Materials

F-graphene was produced and provided by Nanjing Jicang Nano technology Co., Ltd. (Nanjing, China). Bisphenol A diglycidyl ether (E-51 epoxy resin), with an epoxy equivalent of 184, Methyltetrahydrophthalic anhydride (epoxy curing agent), with a relative molecular mass of 168, and dimethylaminomethylphenol (epoxy catalyst) were purchased from Nantong Xingchen Synthetic Material Co., Ltd. (Nantong, China). Other materials, such as anhydrous ethanol, acetone, deionized water, etc., were provided by Chengdu Kelong Chemical Co., Ltd. (Chengdu, China). The use and disposal of all materials in experiments were strictly in accordance with relevant instructions and specifications.

### 2.2. Preparation of F-Graphene/Epoxy Composites

**Figure 1:**
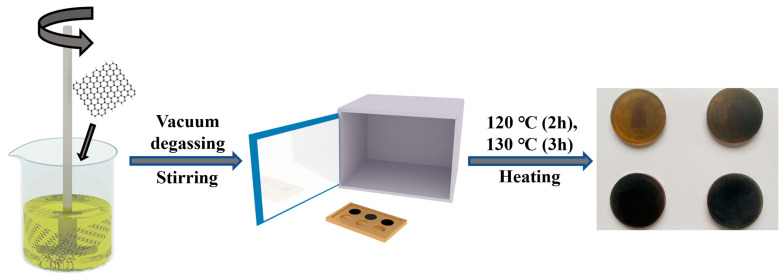
Preparation procedure of the composite material F−graphene/epoxy composites.

F-graphene/epoxy composites were prepared by solid–liquid co-mixing followed by high-temperature curing. Firstly, F-graphene powder was weighed according to different filler mass fractions (0.25 wt.%, 0.50 wt.%, 0.75 wt.%, 1.00 wt.%) and added to 10–15 mL of acetone liquid. The F-graphene/acetone suspension was ultrasonically dispersed at 40 kHz (200 W) for 10 min to obtain a stable dispersion. Then, E-51 epoxy resin, curing agent and catalyst were added to the obtained dispersion at a weight ratio of 100:80:2. The required amount of E-51 epoxy resin was added to the dispersion first, and the mixture was continuously stirred at 400 rpm for 1 h at 50 °C under atmospheric pressure to allow acetone to fully evaporate and F-graphene to disperse well. Then, the curing agent and catalyst were added, and the mixture was further stirred at 450 rpm for 1 h to make the E-51 epoxy resin, curing agent, catalyst and F-graphene filler evenly mixed. Then, the obtained mixture was placed in a vacuum oven, kept at 60 °C and vacuumed (1 bar) for 1 h to remove the large amount of air mixed in during the preparation process. Finally, the degassed mixture liquid was carefully poured into a preheated steel mold (60 °C), placed in a vacuum oven and heated to 80 °C while maintaining vacuum (1 bar) for 1 h to completely remove the residual trace gas in the mixture liquid. Finally, under standard atmospheric pressure, the temperature was raised to 120 °C for 2 h, then continued heating to 130 °C for 2 h and finally cured. After curing and waiting for natural cooling, the composite material was demolded and the surface was polished smooth and flat with fine sandpaper to obtain composite material samples. The preparation procedure of the composite material is shown in Figure 1.

### 2.3. Characterization

The microstructure and morphology of the F-graphene and the characterization of the cross-sections of the composites brittlely fractured by liquid nitrogen (LN_2_) were observed and characterized by scanning electron microscopy (SEM, 5 kV, Zeiss, Gemini300, Oberkochen, BW, Germany). The SEM sample of F-graphene nanostructures was prepared by dispersing the powders in anhydrous ethanol (G.R.) under sonication (80 W) for 5 min and then dropping them onto holey copper grids. Prior to observation, the sample surface for SEM was sputtered with a thin layer of gold. In order to eliminate the sample surface bump caused by the surface tension of liquid epoxy resin during the heat curing process, the surfaces of the samples used for the dielectric properties and thermal conductivity test were sanded flat with 1000 grit sandpaper. The dielectric response of epoxy/F-graphene composites in the frequency range of 10^2^–10^6^ Hz was characterized by a broadband dielectric relaxation spectrometer Concept 50 (Novocontrol Technologies, Montabaur, RP, Germany). The breakdown strength of the samples under a power frequency alternating current (50 Hz) at 25 °C was measured by a ZJC-100 kV computer-controlled voltage breakdown tester (Beijing Zhonghangshidai Instrument Equipment Co., Ltd., Beijing, China). The dynamic thermo-mechanical properties of the composites were analyzed by DMAQ800 (TA Instruments, New Castle, DE, USA), using the three-point bending method in the temperature range of 25–235 °C. The tensile tests of the dumbbell-shaped composite samples at 25 °C were conducted with an Instron 5967 universal material tester (Instron Corporation, Norwood, MA, USA) with a fixture movement speed of 1 mm/min. Thermogravimetric analysis of the samples was performed using a TGA/DSC2 (Mettler-Toledo Technology Co., Ltd., Zurich, Switzerland) in the temperature range of 20~600 °C with a heating rate of 10 °C/min and under a nitrogen atmosphere with a flow rate of 15 mL/min. Considering the differences in absorption and reflection of the laser by the polished surface, the steady-state method was employed to measure the thermal conductivity of the sample. The thermal conductivity of the composites at room temperature (25 °C) was measured by the planar heat flow method (DRL-III, Tan Instrument Co., Xiangtan, China), with high thermal conductivity silver silicone grease applied on the sample surface.

Researchers have proposed a variety of methods for measuring the thermal conductivity of materials, which can be categorized into transient and steady-state methods based on their dependence on the duration of the heating source [37,38,39]. The transient methods employ a periodic or pulsed heat source to study the thermal response of a material as a function of time after it is heated, which include the laser flash method, time-domain thermoreflectance (TDTR) method, 2ω method, photothermal radiation measurement method, etc. Among them, the laser flash method uses laser pulses to heat one side of the sample, measure the temperature rise on the other side and form a functional relationship with time to solve for the thermal conductivity of the sample [40,41,42], while the TDTR employs two ultrafast laser beams, one of which (the pumping light) heats the sample, and the other (the detecting light) samples the temperature change by monitoring the change in reflectivity of the sample surface and compares the temperature change with the appropriate theoretical model to obtain the thermal properties of the sample [43,44]. The 2ω method is a method to measure the thermal response of the sample after heating as a function of time. The 2ω method measures the AC temperature response of the center of a thin film sample to periodic heating by the heat reflection technique and uses a simplified one-dimensional heat conduction equation to measure the transverse thermal conductivity of the film [45,46]. The above transient methods, while having high measurement accuracy, require the researcher to measure the density and specific heat of the sample beforehand, and their extensive use of laser heat sources also puts high demands on the flatness and uniformity of the sample surface. The steady-state methods (such as plane heat flow method, comparative method, etc.), on the other hand, are directly based on Fourier’s law, which determines the thermal conductivity of the measured article by measuring the temperature difference of the sample in the presence of a steady-state heat flow. Among them, the planar heat flow method, as an absolute method of thermal conductivity measurement, has the advantages of simple implementation and high accuracy after controlling errors such as thermal resistance of the contact surface [37,39].

## 3. Results and Discussion

**Figure 2:**
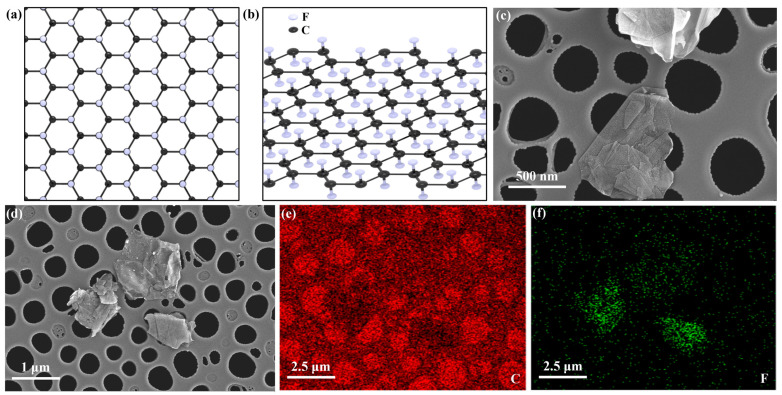
Structural characterization of F−graphene. (**a**) Front view schematic. (**b**) Side view schematic. (**c**) SEM images. (**d**–**f**) SEM EDS mapping.

As shown in Figure 2, the structure of F-graphene used in this study was characterized by scanning electron microscopy (SEM). Figure 2a,b shows the most stable chair-like configuration of F-graphene with a fluorine-carbon ratio of 1:1 [47], where fluorine atoms preferentially bond to the carbon atoms on the opposite side of the graphene layer. The SEM images (Figure 2c) reveal that F-graphene has a lateral size of approximately 2 μm and a very thin thickness, and it exhibits curling, wrinkling and folding features. The edge part of F-graphene also demonstrates its layered characteristic, which is expected to enhance the thermal conductivity and stress strength of the material along the plane direction. As shown in Figure 2d–f, the EDS mapping of carbon and fluorine elements clearly indicates the uniform distribution of fluorine and carbon in F-graphene, confirming the homogeneous fluorination of graphene, which endows it with excellent dielectric properties. Due to the high electronegativity of fluorine atoms, F-graphene possesses good interfacial compatibility, and the abundant -F groups on the surface and edge of its layered structure can form strong C-F-H-F bonds with epoxy molecules, facilitating the formation of nanocomposites.

**Figure 3:**
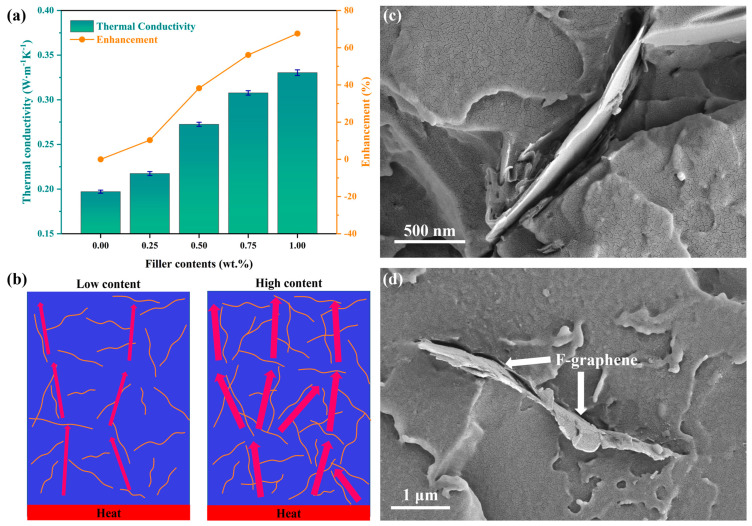
(**a**) Thermal conductivity and enhanced efficiency of F−graphene/epoxy composites. (**b**) Schematic diagram of heat conduction path under different filler contents. (**c**,**d**) SEM images of F−graphene/epoxy composites.

**Figure 4:**
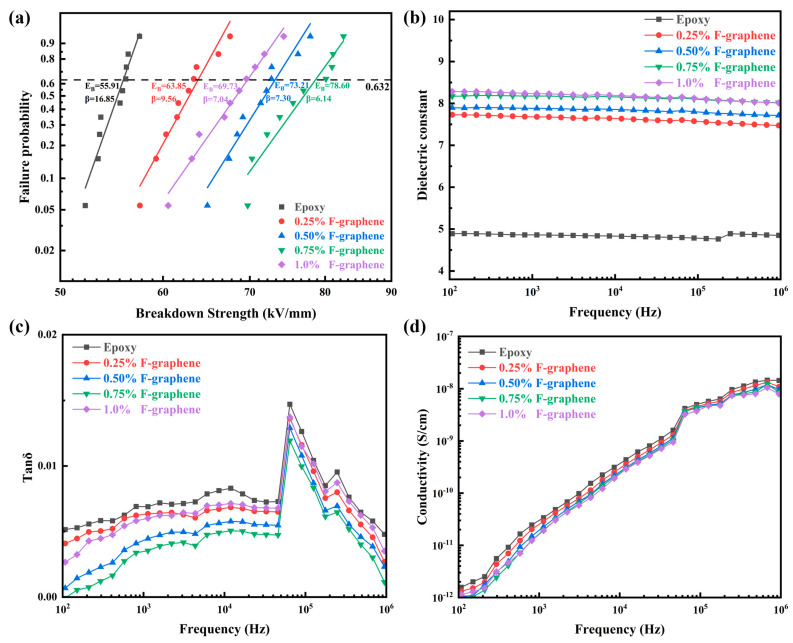
Dielectric properties of F−graphene/epoxy composites at different F−graphene contents (wt.%). (**a**) Double−parameter Weibull plot of the breakdown strength. (**b**−**d**) Dielectric constant, dielectric loss and AC conductivity as a function of frequency.

Figure 3a shows the variation in the thermal conductivity of the composites with the F-graphene content. Thanks to the ultra-high thermal conductivity F-graphene (≥5000 W·m^−1^·K^−1^), the thermal conductivity of the composites achieved a significant improvement compared to pure epoxy resin. As expected, the thermal conductivity of the composites showed a significant positive correlation with the filler content. It is worth noting that at low (≤0.25 wt.%) and high (≥0.75 wt.%) filler contents, the increase in magnitude of the thermal conductivity of the composites was relatively slow. This trend was clearly indicated by the slope of the enhancement rate line in Figure 3a, i.e., the slope of the line segments on both sides was significantly lower than that in the middle part. When the filler content was 0.25 wt.%, the thermal conductivity of the composites was only 0.2174 W·m^−1^·K^−1^, which was 10.30% higher than that of pure epoxy (0.1971 W·m^−1^·K^−1^); when the filler content was 0.5 wt.%, the thermal conductivity of the composites reached 0.2725 W·m^−1^·K^−1^, which was 38.25% higher than that of pure epoxy; and when the filler content was 1.0 wt.%, a thermal conductivity of 0.3304 W·m^−1^·K^−1^ was measured, which was 67.63% higher than that of pure epoxy. This phenomenon can be explained by the structure and random distribution of the filler in the polymer matrix. As illustrated in Figure 3b, F-graphene with a planar structure was mixed in epoxy resin, forming high thermal conductivity “islands” (Figure 3c). However, there is a large difference between the phonon spectra of organic epoxy resin and inorganic F-graphene, resulting in severe phonon scattering at the interface, which causes high interfacial thermal resistance between the two materials, thus being not conducive to cross-interface heat transfer [48,49,50,51]. In this case, when the filler content is low, F-graphene is sparsely distributed in the composites, and the thick epoxy resin layer between them blocks heat flow diffusion, which cannot achieve a significant improvement in thermal conductivity performance. However, when a certain critical amount of filler is added (Figure 3d), the average spacing between F-graphene decreases, even connections occur and heat flow is transferred through an efficient heat conduction path built by high thermal conductivity fillers, thereby significantly increasing the enhancement rate of thermal conductivity [52]. Nevertheless, when the weight percentage of F-graphene is too high (≥0.75 wt.%), severe filler agglomeration may occur, which cannot form a stable and sufficient dispersion in the matrix, and the increase rate of heat conduction channels becomes low, thus limiting the enhanced efficiency of composite thermal conductivity. In addition, a large amount of agglomerated F-graphene filler may also form obvious dielectric defects in the composites, which can be indirectly confirmed by tests on material dielectric properties as shown in Figure 4.

In addition to good thermal conductivity, excellent insulation and dielectric performance are also crucial for the application of polymer dielectrics in electric power and electronic industries. The power frequency (50 Hz) AC breakdown strength of F-graphene/epoxy composites at different filler loadings is shown in Figure 4a, where the two-parameter Weibull distribution function as follows was used to process and analyze the original experimental data [53,54].

(1) $$P\left(E\right)=1-\exp\left(-{\left(\frac{E}{E_{B}}\right)}^{\beta }\right)$$

in which *E* is the experimental breakdown strength obtained from the high-voltage breakdown test; *E*_B_ is the Weibull characteristic breakdown strength, which corresponds to the electric field strength at 63.2% statistical breakdown probability; and the shape parameter *β* indicates the slope of the fitted line and is inversely proportional to the dispersion of the experimental breakdown strength *E*, i.e., the larger *β* is, the steeper the line and the more reliable the Weibull characteristic breakdown strength *E*_B_ is. As shown in Figure 4a, the addition of F-graphene nanofillers enhanced the breakdown strength of all epoxy resin composites. It is worth noting that when the F-graphene content was less than 0.75 wt.%, the breakdown strength increased with the increase in filler content, reaching the peak (78.60 kV/mm, *β* = 6.14) at 0.75 wt.%, which was enhanced to 140.58% of pure epoxy (55.91 kV/mm, *β* = 16.85); but when the filler content reached 1.0 wt.%, the breakdown resistance deteriorated (69.73 kV/mm, *β* = 7.04), and the reliability of the Weibull characteristic breakdown strength (*E*_B_) decreased (i.e., *β* showed a downward trend) with the increase in F-graphene content, which might be affected by the internal insulation defects of the composite materials caused by the incomplete dispersion of F-graphene at high loadings. Thanks to the significantly improved breakdown strength at low filler loadings (0.5 wt.% ~ 0.75 wt.%), the results of this paper have important significance and are expected to meet the high insulation requirements of modern power electronic devices. Furthermore, the employment of F-graphene had a significant impact on the dielectric properties of epoxy resin, as shown in Figure 4b–d. As can be seen from Figure 4b, the dielectric constant of the composites increased with the increase in F-graphene content, and the dielectric constant of pure epoxy resin was 4.86 at 1 kHz frequency, while that of F-graphene/epoxy composites reached 8.23 at an F-graphene loading of 1.0 wt.%, which was about 1.69 times that of pure epoxy. This can be attributed to the interfacial polarization effect introduced by F-graphene nanofiller doping, i.e., because of the significant difference in dielectric properties (e.g., polarity and dielectric constant) between inorganic F-graphene and organic epoxy resin matrix, a high-intensity local electric field is formed at the interface of the two phases, and the free electrons generated within the composite dielectric under the applied electric field are captured at the interface of the two phases, i.e., the charge accumulates in the interfacial region with high field strength, which leads to the increase in space charge within the composites and thus the elevation of the dielectric constant [55,56,57,58,59]. In this study, the interfacial polarization effect can be explained more intuitively by the micro-capacitance model [60,61]. In the composites, F-graphene nanofillers and the epoxy resin matrix can be combined to form a large number of micro-capacitors. Herein, the planar-structured F-graphene can be considered as the electrode plate of the capacitor, the epoxy resin sandwiched between the two ends of the F-graphene electrode plate can be regarded as a polymer dielectric and a strong localized electric field is generated in the micro-capacitor after the application of an electric field. The strong localized electric field generated in the micro-capacitor intercepts a large amount of space charge and improves the dielectric constant of the complex. When the amount of filler is increased, there are more interfaces in the complex, the F-graphene nanofillers are closer to each other and the local field in the thinner filler gap will be further enhanced, which leads to an increase in the magnitude of the interfacial polarization (because a high local field strength can cause more electrons to be trapped at the filler–polymer interface), thus representing an increase in the material’s dielectric constant [62]. This explanation can also be verified by the dielectric constant of the composites at high filler content (≥0.75 wt.%) and high frequency (≥10^5^ Hz) (Figure 4b). The dielectric constant of the composites with 1.0 wt.% loading at low frequency (≤10^5^ Hz) was higher than that with 0.75 wt.% filler loading, while it was reversed when the frequency exceeded about 10^5^ Hz. Such a change in the dielectric constant at high frequency might be attributed to the fact that at this time, the electric field polarity changed too fast, and it was difficult for micro-capacitors formed by interfacial polarization to charge and discharge in time, and this situation became more serious with a higher filler content [63]. As for dielectric loss, F-graphene addition reduced the epoxy dielectric’s dielectric loss (Figure 4c), which would be beneficial for controlling insulation dielectric heating and reducing thermal breakdown probability. This change might originate from F-graphene/epoxy composites being able to capture electrons more efficiently than pure epoxy, thus reducing the possibility of collisions between electrons and matrix molecules and limiting the degree of polarization in alternating electric fields. In addition, the F-graphene doped in the epoxy matrix had extremely low conductivity (<10^−11^ S·cm^−1^), which was conducive to reducing the leakage conduction loss of the obtained dielectric [64], and this point could also be confirmed by the low AC conductivity of the composites (Figure 4d).

**Figure 5:**
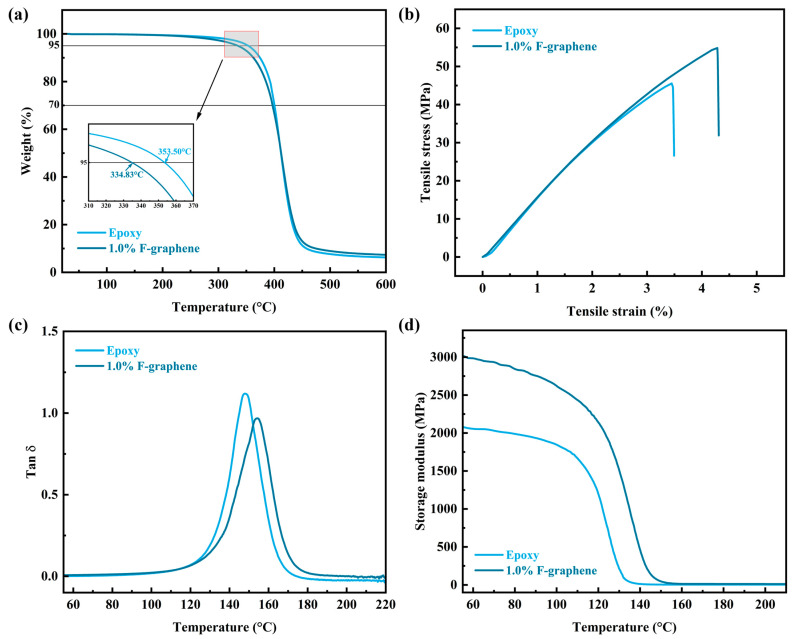
Thermal stability and mechanical properties of F-graphene/epoxy composite. (**a**) TGA curves. (**b**) Stress–strain curves. (**c**,**d**) DMA curves.

Figure 5a shows the TGA (thermogravimetric analysis) curves of epoxy resin and its composites. Generally, the temperature corresponding to 5% weight loss of the sample is regarded as the initial thermal decomposition temperature (*T*_5%_). The *T*_5%_ of neat epoxy is 353.50 °C, while for the composite containing 1.0 wt.% F-graphene, the *T*_5%_ decreases to 334.83 °C, which may be due to the absence of covalent bonds between the nano-inorganic filler and the epoxy matrix. However, at the same time, it can be seen from the decrease of about 5.28% in *T*_5%_ that the introduction of F-graphene does not cause serious adverse effects on the initial thermal decomposition temperature of the epoxy matrix. In addition, the heat resistance performance of the composites can be calculated and measured by the following formula [65].

(2) $$T_{\mathrm{HRI}}=0.49\times \left[T_{5\%}+0.6\times \left(T_{30\%}-T_{5\%}\right)\right]$$

where *T*_HRI_ is the heat resistance index temperature, which characterizes the heat resistance performance of the material; *T*_5%_ is the initial thermal decomposition temperature as mentioned above; and *T*_30%_ is the temperature corresponding to 30% weight loss of the sample. Table 1 shows that there is no significant difference in *T*_HRI_ between the composite and neat epoxy resin, indicating that the doping of F-graphene does not have a negative impact on the heat resistance performance of epoxy composites, which deserves excessive attention.

From the stress–strain curves of tensile tests shown in Figure 5b, it can be seen that the tensile properties of both are basically the same (the curves have the same characteristics), but the composite is more tough than neat epoxy resin, which is reflected in the composite’s ability to withstand higher tensile strain. This may be because the F-graphene nanofiller dispersed in the matrix can effectively enhance the crosslinking degree of epoxy resin molecules, thereby increasing the material’s strain limit [14]. Moreover, the enhancement of the mechanical properties of the composites is also supported by dynamic thermomechanical analysis (DMA), which shows that the glass transition temperature (*T*_g_ = 154.19 °C) of the composites is higher than that of pure epoxy (*T*_g_ = 147.84 °C) (Figure 5c), and the storage modulus is also significantly higher than that of pure epoxy (Figure 5d). This can also be attributed to the increased molecular crosslinking degree. Because it is a thermosetting resin, the molecular crosslinking density (υ) of epoxy resins and their composites can be calculated based on the elasticity theory from the storage modulus measured by DMA using the following formula [66,67].

(3) $$\upsilon =\frac{G}{RT}$$

where *G* is the shear modulus of the polymer, *R* is the gas constant and *T* is the thermodynamic temperature (K). *G* in Equation (3) can be obtained from Equation (4)

(4) $$G=\frac{E}{2\left(1+V\right)}$$

in which *E* is the modulus of elasticity, while *V* is the Poisson’s ratio of the material, for epoxy resin *V* takes the value of 0.38. After substituting the value of *V* into Equation (4) and further bringing it into Equation (3), we can obtain the crosslinking density calculation Formula (5)

(5) $$\upsilon =\frac{E}{2.76RT}$$

Here, the value of the elastic modulus *E* can be equated with the storage modulus *E*’ at low frequencies. The low-frequency storage modulus *E* of the sample, the temperature *T* and the calculated crosslink density υ are shown in Table 2. Compared with pure epoxy, the higher molecular crosslinking density of the composites ($1.19\times 10^{-3\;}\mathrm{mol}/\mathrm{cc}$) can effectively restrict the movement of epoxy resin molecular chains and reduce intermolecular friction and energy loss, thus achieving the enhancement of *T*_g_ and material stiffness (i.e., storage modulus increase).

## 4. Conclusions

In conclusion, by introducing F-graphene nanofillers, which combine the advantages of high thermal conductivity, wide bandgap, low conductivity and high stability, into the development of epoxy-based polymer dielectrics, a nanocomposite dielectric with excellent performance was successfully fabricated in this study, and the synergistic optimization of the important characteristics was effectively achieved. Thanks to the extremely high thermal conductivity and planar structure of F-graphene, efficient and robust heat transfer channels were constructed in the F-graphene/epoxy composite, resulting in a thermal conductivity of 0.3304 W·m^−1^·K^−1^ for the composite. The high resistivity of F-graphene enhanced the breakdown resistance and AC resistivity of the composite material. The abundant interfaces caused by the nanofillers introduced considerable interfacial polarization effects, which increased the dielectric constant of the composite and reduced the dielectric loss by trapping electrons. Moreover, the crosslinking of epoxy resin molecules became more compact with the addition of F-graphene, which improved the mechanical performance of the composite. Benefiting from the excellent thermal conductivity and insulating and dielectric properties, the F-graphene/epoxy composite provided in this study is expected to be widely used as a thermal management material or insulating and dielectric medium in the present-day advanced power electronic devices and integrated devices with high heat generation density and high integration. Its excellent mechanical properties and stability will also boost the application of this composite in the field of power equipment packaging and support, such as for grid reactors. The preparation method of blending the filler with the epoxy matrix has the advantage of simple and convenient preparation while avoiding the possibility of environmental pollution by surface modifiers and the like. Herein, we strongly believe that the results of this study provide a meaningful reference for the preparation of epoxy composite dielectrics with outstanding thermal conductivity and dielectric properties.

## Figures and Tables

**Table 1:** TGA characteristic temperatures of epoxy resin and its F-graphene composites.

Samples	Weight Loss Temperature (°C)		*T*_HRI_ (°C)
	${T_{5\%}}_{\;}$	${T_{30\%}}_{\;}$	
Neat epoxy resin	353.50	400.33	186.98
1.0 wt.% F-graphene/epoxy	334.83	396.95	182.33

**Table 2:** Physical parameters measured by DMA and calculated crosslink densities of epoxy resin and its F-graphene composites.

Samples	Physical Parameters		*υ* (10^−3^mol/cc)
	*E*′ (MPa)	*T* (K)	
Neat epoxy resin	3.21	450.99	0.31
1.0 wt.% F-graphene/epoxy	12.45	457.34	1.19

## Data Availability

The raw data presented in this study are available on request from the corresponding author, without undue reservation.
